# Pandemic clinical case definitions are non-specific: multiple respiratory viruses circulating in the early phases of the 2009 influenza pandemic in New South Wales, Australia

**DOI:** 10.1186/1743-422X-11-113

**Published:** 2014-06-18

**Authors:** Vigneswary Mala Ratnamohan, Janette Taylor, Frank Zeng, Kenneth McPhie, Christopher C Blyth, Sheena Adamson, Jen Kok, Dominic E Dwyer

**Affiliations:** 1 Centre for Infectious Diseases and Microbiology Laboratory Services, Institute of Clinical Pathology and Medical Research, Westmead Hospital, Westmead, New South Wales, Australia; 2 Centre for Virus Research, Westmead Millennium Institute, Westmead Hospital, Westmead, New South Wales, Australia; 3 School of Paediatrics and Child Health, University of Western Australia, Crawley, Western Australia, Australia; 4 Department of Paediatrics and Adolescent Medicine and PathWest Laboratory Medicine WA, Princess Margaret Hospital, Subiaco, Western Australia, Australia; 5 Communicable Diseases Branch, Health Protection New South Wales, New South Wales Health, North Sydney, New South Wales, Australia; 6 Marie Bashir Institute for Infectious Diseases and Biosecurity, University of Sydney, Westmead Hospital, Westmead, New South Wales, Australia; 7 Centre for Research Excellence in Critical Infections, University of Sydney, Westmead Hospital, Westmead, New South Wales, Australia

**Keywords:** Influenza, A(H1N1)pdm09, Pandemic, Respiratory viruses

## Abstract

**Background:**

During the early phases of the 2009 pandemic, subjects with influenza-like illness only had laboratory testing specific for the new A(H1N1)pdm09 virus.

**Findings:**

Between 25^th^ May and 7^th^ June 2009, during the pandemic CONTAIN phase, A(H1N1)pdm09 virus was detected using nucleic acid tests in only 56 of 1466 (3.8%) samples meeting the clinical case definition required for A(H1N1)pdm09 testing. Two hundred and fifty-five randomly selected A(H1N1)pdm09 virus-negative samples were tested for other respiratory viruses using a real-time multiplex PCR assay. Of the 255 samples tested, 113 (44.3%) had other respiratory viruses detected: rhinoviruses 63.7%, seasonal influenza A 17.6%, respiratory syncytial virus 7.9%, human metapneumovirus 5.3%, parainfluenzaviruses 4.4%, influenza B virus 4.4%, and enteroviruses 0.8%. Viral co-infections were present in 4.3% of samples.

**Conclusions:**

In the very early stages of a new pandemic, limiting testing to only the novel virus will miss other clinically important co-circulating respiratory pathogens.

## Short report

The emergence of a novel pathogen of pandemic potential, as occurred in 2009 with the influenza A(H1N1)pdm09 virus, requires the development of clinical case definitions to assist in managing the clinical, laboratory and public health responses to the new disease. A(H1N1)pdm09 virus is a quadruple reassortant virus of mostly swine-origin influenza genes that was first reported in the United States of America (USA) on 24^th^ April 2009 [1], although it was believed to have emerged in Mexico earlier [2].

The Australian Health Management Plan for Pandemic Influenza (AHMPPI) [3], was executed prior to the declaration of pandemic influenza by the World Health Organisation on the 11^th^ June 2009. Specific testing for A(H1N1)pdm09 virus began on the 28^th^ April at the start of the ALERT phase. The ALERT phase was defined as the period when only a few cases of A(H1N1)pdm09 had been reported in a few countries, but not in Australia, with control measures implemented for quarantine purposes [3]. The CONTAIN phase defined the period when the A(H1N1)pdm09 virus had arrived in Australia with few confirmed infections or small clusters of cases, with measures in place for the rapid identification of infections and containment [3]. The first Australian cases were imported from the Americas; the first locally-acquired case in New South Wales (NSW) was identified on the 20^th^ May: a 3 year old whose mother had acquired laboratory-confirmed A(H1N1)pdm09 virus infection during a 10-day holiday to the USA [4]. During the CONTAIN phase between 22^nd^ May and 16^th^ June, all laboratory requests were triaged to limit testing to individuals who met the CONTAIN phase clinical case definition: individuals showing symptoms of influenza-like illness (ILI) within 7 days of travel to A(H1N1)pdm09-declared areas, or people with febrile respiratory illness with onset within 7 days of contact with a laboratory-confirmed case [5]. Although clinical case definitions may be sensitive for a newly circulating pandemic virus, it may lack specificity, and testing algorithms detecting the novel virus alone will fail to detect other co-circulating respiratory viruses.

Within Australia during the early pandemic phases, the cities of Melbourne and Brisbane reported cases earlier than Sydney (NSW). Following the first laboratory-confirmed A(H1N1)pdm09 case in NSW, another six influenza A cases were reported on the 24^th^ May from passengers of a cruise ship that had departed Sydney on a 10-day cruise in the Pacific Ocean on the 16^th^ May. The ship stopped at New Caledonia and Vanuatu, islands that had not reported circulation of A(H1N1)pdm09 virus at the time [6]. Combined nose and throat swabs collected in viral transport medium were tested for A(H1N1)pdm09 virus by nucleic acid tests (NAT) at the WHO National Influenza Centre in Sydney. Between the 25^th^ May and the 7^th^ June, 1466 patient samples met the CONTAIN-phase definition for laboratory testing, of which 56 (3.8%) were positive for A(H1N1)pdm09 virus by NAT. Of these, 43/56 reported a travel history outside Australia, including the 37 passengers on the cruise ship and six with a history of air travel to other countries. From the 4-7^th^ June, 13 locally acquired cases from known contacts were identified, 11 from contacts within Australia and two from people who had recently returned from the USA. Samples received from the 25^th^ May to 7^th^ June were considered representative of the population suffering from ILI not requiring hospitalisation. As A(H1N1)pdm09 virus was only detected in 3.8% of samples, we performed NAT for other respiratory viruses on randomly selected samples collected from subjects that had met the clinical case definition during the CONTAIN phase.

Following A(H1N1)pdm09 virus-specific NAT as previously described [7], further NAT for other respiratory viruses was performed on residual cDNA or extracted total nucleic acid (NA) stored at either −20°C or −70°C. The cDNA was prepared from NA primed with random hexamers using Superscript III RT enzyme (InVitrogen Life Technologies, Carlsbed, CA, USA), according to the manufacturer’s protocol. NAT was carried out on the Roche LC 480 real-time instrument (Roche Diagnostics GmbH, Mannheim, Germany) in a multiplex format, with influenza A, influenza B and respiratory syncytial virus (RSV) targets in one primer set; parainfluenzaviruses 1, 2 and 3 in the second set; and human metapneumovirus (hMPV), rhinoviruses and enteroviruses in the third set. Human adenoviruses and a house-keeping gene, beta-globin (used as a sample inhibition control) [8], were tested directly from the NA extract and formed the fourth set.

Four μLs of cDNA or extract was amplified in a total volume of 20 μL with 0.5 μM of each primer and 0.2 μM of each probe and 10 μL of Lightcycler Probes Master (Roche Diagnostics GmbH) in the LC480 microwell plate. Primers and probes were designed in-house or chosen from previous publications [9-11] and optimised so that all targets were efficiently amplified under the same conditions. The assay was evaluated against a panel of known positive and negative respiratory specimens [12]. To increase the sensitivity of detection of the commonly known serotypes of rhinoviruses, enteroviruses and adenoviruses, two sets of primers and/or 2 probes were used for each of the virus groups (Table 1). The Taqman real-time PCR assay conditions consisted of denaturation (95°C - 10 min) and amplification (40 cycles of 95°C - 10 sec, 58°C −15 sec, 72°C - 20 sec).

**Table 1:** Primer and probe sequences used in the respiratory virus multiplex assays

**Virus (target gene)**	**Name**	**Sequence (5′ to 3′ end)**	**Reference**
RSV (nucleoprotein)	RSVF	For: tagtgtrcargcagaaatgg	in-house
	RSVAR	Rev: agtgrggaaattgagtcaaagat	in-house
	RSVBR	Rev: rggraattgagttaatgacagc	in-house
	RSVP	Probe: FAM tgatgcttttggrttrttcaatatatgg BHQ1	in-house
Influenza A (matrix)	AMF2	For: atggaatggctaaagacaagac	[9]
	AMR2	Rev: cattkagggcattytggac	in-house
	AMP	Probe: Cal fluo Red610 acgctgcagtcctcgctcact BHQ2	in-house
Influenza B (nucleoprotein)	BNF	For: yaacgatgacatggagagaaac	in-house
	BNR	Rev: gcctcctgttttgttgtgatc	in-house
	BNP	Probe: Quasor670 ccttctttsacatctctggcattctt BHQ2	in-house
Parainfluenza 1 (matrix)	PI1F	For: yggaacatcactaggtacaatyac	in-house
	PI1R	Rev: gagcttcttttctccatcatc	in-house
	Para1P	Probe: aactcttgcagatcttgcattaccg	in-house
Parainfluenza 2 (matrix)	PI2F	For: tcactgtggtcagttggatgt	in-house
	PI2R	Rev: ctcaaatgtccgttgacctg	in-house
	Para2P	Quasor670 aagaatctgatcttaatgagctaatgggc BHQ2	in-house
Parainfluenza 3 (matrix)	PI3F	For: Cal fluo Red610 gaagtgagaagaacagtyaaagc BHQ2	in-house
	PI3R	Rev: cattgaggagcaagagcaac	in-house
	Para3P	Probe FAM ttggcatcgaacarcattcc BHQ1	in-house
Rhino & Entero (5′UTR)	Rhi3A	For: gcccctgaatgyggctaa	[10]
	Rhi4B	Rev: gaaacacggacacccaaagta	[10]
	Rhi1P	Probe: FAM tggtcccrtcccgcamttgc BHQ1	in-house
	Rhi2P	Probe: FAM ccrtcccrsaattgctcrttacgac BHQ1	in-house
	Ent1P	Probe: Cal fluo Red610 cggttccgctgcrgagttrccc BHQ2	in-house
	Ent2P	Probe: Cal fluo Red610 cggttccgccacrgacttrcgc BHQ2	in-house
hMPV (nucleoprotein)	Meta NLNF	For: catayaarcatgctatattaaaagagtctc	[11]
	Meta NLNR	Rev: cctatytctgcagcatatttgtaatcag	[11]
	MetaP	Probe:Quasor670 tcttgytgcaatgatgarggtgtyactgc BHQ2	[11]
Adeno (hexon)	AdeF	For: caaggaygtmaacatgatcctgcag	in-house
	AdeF	For: raaggatgtdaacatgrtbctdcag	in-house
	AdeR	Rev: cgttggtrtcgttrcgcagcat	in-house
	AdeR	Rev: crttggtrtcrttycknarcat	in-house
	Ade1P	Probe: FAM trgaagckgtgttrtgwgccatggg BHQ1	in-house
	Ade2P	Probe: FAM tggaggcsgtgttgtgsgccatggg BHQ1	in-house
Human Beta-globin	PCO3	For: acacaactgtgttcactagc	[8]
	PCO4	Rev: caacttcatccacgttcacc	[8]
	BGLP	Probe: Quasor670 tcaaacagacaccatggtgcacctga BHQ2	in-house

Clinical information was gathered from individual test request forms and NetEpi, the NSW Health surveillance system; data was unavailable for 38 individuals. As the clinical case definition was strictly applied in the early CONTAIN phase, we assume that all subjects without any clinical information recorded on the request form met the testing criteria. Results are shown in Table 2. Of 255 samples tested (collected from 219 adults, 33 children and 3 with no age recorded), 113 (44.3%) had one or more respiratory viruses detected: 72 (63.7%) rhinoviruses, 20 (17.6%) seasonal influenza A viruses (16 A/H3N2 and 4 A/H1N1), 9 (7.9%) RSV, 6 (5.3%) hMPV, 5 (4.4%) each of influenza B and parainfluenzaviruses and 1 (0.8%) enterovirus. Five viral co-infections were identified, four with RSV and rhinoviruses, and one with influenza A/H3N2 and rhinovirus. No human adenoviruses were detected. The respiratory virus detection rate in the paediatric sub-group was 63.6% (21/33 tested), with 12 rhinoviruses, 3 influenza A/H3N2, 2 RSV, 1 parainfluenzavirus 2, 1 enterovirus and 2 co-infections with RSV and rhinoviruses.

**Table 2:** Detection rates of respiratory viruses (*denotes co-infection)

**Category of patients with influenza-like illness**	**Number tested**	**RSV**	**Influenza A**	**Influenza B**	**Parainfluenza 1**	**Parainfluenza 2**	**Parainfluenza 3**	**Rhinovirus**	**Enterovirus**	**hMPV**	**Adenovirus**	**None detected**
From Americas	40	1 (+RV*)	1 H3	1	0	0	0	13	0	3	0	22
Cruise ships	77	0	5 H3	0	0	0	0	20	1	1	0	50
Western Pacific Region (Fiji, Vanuatu, N Zealand, Singapore, China, H Kong, Philippines)	9	0	2 H3	0	0	0	0	3	0	0	0	4
From Europe	12	1	1 H3	0	0	0	0	5	0	0	0	5
South East Asia Region (Indonesia, Thailand, S Korea, India)	7	1 (+RV*)	1 H1	0	0	0	1	2	0	0	0	3
From Japan	5	0	0	0	0	0	0	1	0	0	0	4
From Africa (Ethiopia)	1	0	0	0	0	0	0	1	0	0	0	0
Interstate travel (Melbourne)	16	0	2 H3	0	0	1	0	3	0	1	0	9
No travel, but in contact with travellers, confirmed or suspected	20	1	0	3	0	0	0	5	0	1	0	10
Overseas travel, location unknown	3	0	0	0	0	0	0	2	0	0	0	1
No record of travel or contact, but record of clinical symptoms	27	3 (paediatric)	4 (3 H3, 1 H1)	0	0	1	0	7 (1 + RSV*)	0	0	0	13
Unknown -no clinical data provided	38	2	4 (2 H3,2 H1)	1	0	0	2	10 (1 + RSV*) (1 + H3*)	0	0	0	21
TOTAL	255	9	20 (16 H3 and 4 H1)	5	0	2	3	72	1	6	0	142
Number of patients with a viral infection = 113												
Number of patients with 2 viral co-infections = 5												

In the present study, despite limiting laboratory testing to those meeting the clinical case definition, A(H1N1)pdm09 virus was only detected in 3.8% of cases during the early CONTAIN phase; rhinoviruses instead were the most commonly detected virus. Although the samples were collected from subjects in an outpatient setting in this study, rhinoviruses have also been identified as the most common virus detected in hospitalised children presenting with an acute respiratory infection [13]. Outbreaks of rhinovirus infection, sometimes causing severe disease, have also been reported in long-term care facilities and in elderly adults presenting with respiratory distress to hospital emergency departments [14,15]. In another study determining the rate of non-A(H1N1)pdm09 virus infection during the 2009 pandemic at two hospital emergency departments in France, rhinovirus infection rates were also higher than A(H1N1)pdm09 virus infection rates (62.6% vs. 16.5%) [16]. In our study, the rate of rhinovirus infection in travellers and contacts of cases was similar. Other respiratory viruses contributed approximately 20% of infections. Viral co-infections were also uncommon, possibly from sampling bias as viral co-infection may be associated with more severe disease.

Clinical syndromes defining typical influenza virus infection overlap with those described for other respiratory viruses. Non-specific case definitions, along with public health, media and clinical demands (given that the first pandemic wave in 2009 occurred during the usual influenza and respiratory virus season), may contribute to unrealistic pressures on laboratories. Using a broad clinical case definition (even when limiting it only to travellers) in the early phases of a new pandemic or following emergence of other novel respiratory viruses overseas, as exemplified by the recent emergence of the Middle East Respiratory Syndrome-coronavirus [17] is non-specific. Pathogen-specific NAT using a sensitive, but non-specific case definition targeted to a new pathogen is needed in a containment phase for management of infected patients and related public health issues. However, this approach will miss many other respiratory viruses that in themselves may have clinical significance in certain populations, even if not hospitalised.

Even prior to the widespread transmission of A(H1N1)pdm09 virus in Australia, limiting testing to travellers did not improve the specificity of testing. Furthermore, if laboratories use NAT to determine other causes of infection, testing capacity in an outbreak may soon be reached. However, when the causative pathogen of an outbreak has been identified and the outbreak has progressed beyond containment, then the testing algorithms need revision to target only specific indications, such as a location of new or significant clusters, or for individuals at risk of severe disease.

In conclusion, laboratory testing specifically targeting only the new virus will miss other clinically important co-circulating respiratory pathogens in the very early stages of a pandemic. Detecting the presence of other viruses may provide important information on the impact of pre-existing viruses when a new pandemic virus is circulating.

## Competing interests

The authors declare that they have no competing interests.

## Authors’ contributions

VMR participated in the design of the study, design of primers and drafted the manuscript. JT was involved with clinical data management during the 2009 influenza A H1N1 pandemic. FZ performed NAT testing. KM was involved in laboratory sample testing and management. CCB had clinical input on testing strategy. SA was the coordinator of the NSW laboratory response. JK edited the manuscript. DED participated in the design of the study and was involved in the manuscript editing. All authors have read and accepted the manuscript.

## References

[B1] Dawood FS Jain S Finelli L Shaw MW Lindstrom S Garten RJ Gubareva LV Xu X Bridges CB Uyeki TM Emergence of a novel swine-origin influenza A (H1N1) virus in humans N Engl J Med 2009 360 2605 2615 19423869 10.1056/NEJMoa0903810

[B2] Perez-Padilla R de la Rosa-Zamboni D Ponce de Leon S Hernandez M Quiñones-Falconi F Bautista E Ramirez-Venegas A Rojas-Serrano J Ormsby CE Corrales A Higuera A Mondragon E Cordova-Villalobos JA for the INER Working Group on Influenza Pneumonia and respiratory failure from swine-origin influenza A (H1N1) in Mexico N Engl J Med 2009 361 680 689 10.1056/NEJMoa0904252 19564631

[B3] Australian Health Management Plan for Pandemic Influenza [http://www.health.gov.au/internet/panflu/publishing.nsf/Content/B11402BB723E0B78CA25781E000F7BB/$File/ahmppi-2009.pdf]

[B4] Spokes PJ Cretikos MA Ward JG Pandemic (H1N1) 2009 influenza in NSW: an overview of the public health response N S W Public Health Bull 2010 21 4 9 10.1071/NB09035 20374687

[B5] Churches T Conaty SJ Gilmour RE Muscatello DJ Reflections on public health surveillance of pandemic (H1N1) 2009 influenza in NSW N S W Public Health Bull 2010 21 19 25 10.1071/NB09046 20374690

[B6] Ward KA Armstrong P McAnulty JM Iwasenko JM Dwyer DE Outbreaks of pandemic (H1N1) 2009 and seasonal influenza A (H3N2) on cruise ship Emerg Infect Dis 2010 16 1731 1737 10.3201/eid1611.100477 21029531 PMC3294517

[B7] Kok J Blyth CC Foo H Patterson J Taylor J McPhie K Ratnamohan VM Iredell JR Dwyer DE Comparison of a rapid antigen test with nucleic acid testing during co-circulation of pandemic influenza A/H1N1 2009 and seasonal influenza A/H3N2 J Clin Microbiol 2010 48 290 291 10.1128/JCM.01465-09 19889892 PMC2812250

[B8] Saiki RK Scharf S Faloona F Mullis KB Horn GT Erlich HA Arnheim N Enzymatic amplification of beta-globin genomic sequences and restriction sites analysis for analysis of sickle cell anemia Science 1985 230 1350 1354 10.1126/science.2999980 2999980

[B9] Scheweiger B Zadow I Heckler R Timm H Pauli G Application of a fluorogenic PCR assay for typing and subtyping of influenza viruses in respiratory samples J Clin Microbiol 2000 38 1552 1558 10747142 10.1128/jcm.38.4.1552-1558.2000 PMC86487

[B10] Jokela P Joki-Korpela P Maaronen M Glumoff V Hyypiä T Detection of human picornaviruses by mutliplex reverse transcription-PCR and liquid hybridization J Clin Microbiol 2005 43 1239 1245 10.1128/JCM.43.3.1239-1245.2005 15750090 PMC1081250

[B11] Maertzdorf J Wang CK Brown JB Quinto JD Chu M de Graaf M van den Hoogen BG Spaete R Osterhaus AD Fouchier RA Real-Time Reverse Transcriptase PCR assay for detection of human metapneumoviruses from all known genetic lineages J Clin Microbiol 2004 42 981 986 10.1128/JCM.42.3.981-986.2004 15004041 PMC356857

[B12] MacIntyre CR Ridda I Seale H Ratnamohan VM Donovan L Zeng F Dwyer DE Respiratory viruses transmission from children to adults within a household Vaccine 2012 30 3009 3014 10.1016/j.vaccine.2011.11.047 22119589 PMC7115576

[B13] Cheuk DK Tang IW Chan KH Woo PC Peiris MJ Chiu SS Rhinovirus infection in hospitalized children in Hong Kong: a prospective study Pediatr Infect Dis J 2007 26 995 1000 10.1097/INF.0b013e3181586b63 17984805

[B14] Longtin J Marchand-Austin A Winter AL Patel S Eshaghi A Jamieson F Low DE Gubbay JB Rhinovirus outbreaks in long-term care facilities, Ontario, Canada Emerg Infect Dis 2010 16 1463 1465 10.3201/eid1609.100476 20735934 PMC3294989

[B15] Pierangeli A Scagnolari A Selvaggi C Verzaro S Spina MT Bresciani E Antonelli G Bertazzoni G Rhinovirus frequently detected in elderly adults attending an emergency department J Med Virol 2011 83 2043 2047 10.1002/jmv.22205 21915880 PMC7166537

[B16] Schnepf N Resche-Rigon M Chaillon A Scemla A Gras G Semoun O Taboulet P Molina JM Simon F Goudeau A LeGoff J High burden of non-influenza viruses in influenza-like illness in the early weeks of H1N1v epidemic in France PLoS One 2011 6 e23514 10.1371/journal.pone.0023514 21858150 PMC3157400

[B17] Corman VM Eckerle I Bleicker T Zaki A Landt O Eschbach-Bludau M van Boheemen S Gopal R Ballhause M Bestebroer TM Muth D Müller MA Drexler JF Zambon M Osterhaus AD Fouchier RM Drosten C Detection of a novel human coronavirus by real-time reverse-transcription polymerase chain reaction Euro Surveill 2012 17 20285 23041020 10.2807/ese.17.39.20285-en

